# Analysis of the uterine artery pulsatility index on the day of endometrial transformation and pregnancy outcomes of patients undergoing frozen–thawed embryo transfer

**DOI:** 10.3389/fendo.2024.1278504

**Published:** 2024-04-15

**Authors:** Li Li, Mingze Du, Sheling Wu, Caiyuzhu Wen, Pingping Kong, Junwei Zhang, Yichun Guan

**Affiliations:** The Reproductive Center, The Third Affiliated Hospital of Zhengzhou University, Zhengzhou, Henan, China

**Keywords:** frozen-thawed embryo transfer, endometrial transformation day, uterine artery, pulsation index, pregnancy outcome

## Abstract

**Objective:**

The objective was to analyze the impact of the uterine artery pulsatility index (PI) on pregnancy outcomes by measuring uterine artery blood flow on the day of endometrial transformation in patients undergoing frozen–thawed embryo transfer (FET).

**Methods:**

This was a case-control study. In total, 2,036 patients who underwent FET at the Third Affiliated Hospital of Zhengzhou University from October 2019 to September 2020 were included. The patients were divided into a clinical pregnancy group and a nonclinical pregnancy group according to pregnancy outcome. A multivariate logistic regression model was used to analyze the factors affecting the clinical pregnancy rate. The receiver operating characteristic (ROC) curve was used to determine the optimal mean PI cutoff value of 1.75. After 1:1 propensity score matching (PSM), 562 patients were included. For statistical description and analysis, the patients were divided into two groups: a group with a mean PI > 1.75 and a group with a mean PI ≤ 1.75.

**Results:**

The clinical pregnancy group included 1,218 cycles, and the nonclinical pregnancy group included 818 cycles. There were significant differences in female age (P<0.01), infertility type (P=0.04), baseline follicle-stimulating hormone level (P=0.04), anti-Müllerian hormone (AMH) level (P<0.01), antral follicle count (P<0.01), number of transferred embryos (P=0.045) and type of transferred embryo (P<0.01). There was no significant difference in the mean bilateral PI (1.98 ± 0.34 vs. 1.95 ± 0.35, P=0.10). The multivariate analysis results showed that maternal age (AOR=0.95, 95% CI=0.93-0.98, P<0.01), AMH level (AOR=1.00, 95% CI=1.00-1.01, P=0.045), number of transferred embryos (AOR=1.98, 95% CI=1.47-2.70, P<0.01), and type of transferred embryo (AOR=3.10, 95% CI=2.27-4.23, P<0.01) were independent factors influencing the clinical pregnancy rate. The mean PI (AOR=0.85, 95% CI=0.70-1.05; P=0.13) was not an independent factor influencing the clinical pregnancy rate. Participants were divided into two groups according to the mean PI cutoff value of 1.75, and there was no significant difference between the two groups (P > 0.05).

**Conclusion:**

In this study, we found that the uterine artery PI on the day of endometrial transformation in patients undergoing FET is not a good predictor of pregnancy outcomes.

## Introduction

The incidence of infertility is reported to be 15-20% (1). Over the past 30 years, the emergence of *in vitro* fertilization–embryo transfer (IVF–ET) technology has revolutionized the treatment of infertility, and this method has become the ultimate choice for various conditions related to infertility. Embryo quality and endometrial receptivity are important factors determining the success of assisted reproductive treatment, and endometrial receptivity is an important link in ensuring the implantation of fertilized eggs and normal fetal and placental development. However, over the years, with improvements in technologies such as controlled ovarian hyperstimulation (COH), *in vitro* fertilization, and embryo culture, the quantity and quality of embryos have gradually improved. However, the IVF–ET pregnancy rate is approximately 40%, and research on endometrial receptivity is increasingly valued by scholars both domestically and internationally. Endometrial receptivity (2) refers to the ability of the endometrium to allow for the normal implantation of an embryo. Current methods for evaluating endometrial receptivity include endometrial receptivity markers (such as pinopodes), endometrial receptivity factors (such as interleukin, epidermal growth factor, and vascular endothelial growth factor levels, estrogen receptors and progesterone receptors) and ultrasound. Ultrasound is a fast, economical, noninvasive and repeatable dynamic examination method. For infertile patients, transvaginal ultrasound can be used not only to better understand the conditions of the uterus, ovaries and pelvic cavity but also to check uterine artery blood flow through color Doppler and energy Doppler. With the wide application of transvaginal color Doppler ultrasound in the field of reproductive medicine, many scholars have begun to study the relationship between uterine artery blood flow and pregnancy outcomes. In 1988, Goswamy et al. (3) proposed for the first time that uterine artery blood perfusion insufficiency might be the cause of pregnancy failure and suggested that endometrial receptivity could be understood by monitoring uterine artery blood flow with Doppler ultrasound to improve treatment plans and increase the pregnancy rate. Uterine artery blood flow plays an important role in pregnancy success (4). The PI, calculated as PI=[peak systolic flow velocity (A) - diastolic flow velocity (B)]/average flow velocity], reflects not only the peak systolic flow velocity and diastolic flow velocity but also the average flow velocity of the entire cardiac cycle and represents the overall situation of the blood flow waveform. Therefore, the PI can better reflect the resistance of uterine artery blood vessels and has greater clinical significance.

In this study, we measured and analyzed the relationship between the uterine artery pulsatility index (PI) and pregnancy outcomes.

## Methods

### Population

This was a retrospective cohort study that was approved by the review board of the Third Affiliated Hospital of Zhengzhou University (2022-204-01). The study involved women who underwent frozen–thawed embryo transfer (FET) cycles at the Reproductive Medicine Department of the Third Affiliated Hospital of Zhengzhou University from October 2019 to September 2020. Women aged <40 years were included. Women with chromosomal abnormalities, congenital uterine dysplasia, intrauterine adhesions, hydrosalpinx, comorbid medical diseases (hypertension, diabetes, thyroid dysfunction, liver dysfunction, thrombocytopenia, etc.), or comorbid acute hemorrhagic diseases (peptic ulcer, etc.) or who were missing important data in their cycle records were excluded.

### Embryo transfer and endometrial preparation protocols

#### Program and drug support

In FET cycles, different FET schemes were formulated according to the specific conditions of each patient. Endometrial thickness, follicle development and serum hormone levels were continuously monitored. The endometrium was transformed in a timely manner, and 1 to 2 cleavage-stage embryos were transferred 3 days after endometrial transformation; alternatively, 1 blastocyst was transferred 5 days after endometrial transformation.

Endometrial preparation for FET was performed by means of the natural cycle for women with regular menstrual cycles and spontaneous ovulation; artificial/induced ovulation cycles were used for women with irregular menstrual cycles; and downregulation and artificial cycles were used for women with endometriosis.

#### Natural/induced ovulation cycle

Ovulation monitoring began on day 10 of the menstrual cycle for patients with a normal menstrual cycle, and ovulation was induced by oral letrozole on day 3 of the cycle for patients with irregular or anovulatory cycles.

Outcome measures and definitions: Serum luteinizing hormone (LH), estradiol (E_2_) and progesterone (P) levels were measured when the dominant follicle diameter was greater than 16 to 18 mm and the endometrial thickness was at least 7 mm. When the E_2_ level was greater than 100 pg/mL and the P level was less than 1.5 ng/mL, beta-human chorionic gonadotropin (β-HCG; Lizhu Pharmaceutical Trading, China) 10000 (IM) was injected intradurally according to the LH level. Two days later, P was converted to oral dydrogesterone (Dapotone, Solvay Pharmaceuticals, Netherlands; 30 mg/day), and P sustained-release gel (Ceronotone, Merck Serono, Switzerland; 90 mg/day) was administered vaginally. After 3 or 5 days of progesterone conversion in the endometrium, frozen-thawed cleavage-stage embryos or frozen-thawed blastocysts were transferred by abdominal ultrasound monitoring; 14 days after transplantation, venous blood was drawn to determine the β−hCG concentration. The blastocyst transfer outcome was determined after 30 days by vaginal ultrasound. If pregnancy occurred, luteal support was continued until at least 65 days after transfer (5).

#### Artificial cycle

Vaginal ultrasound was performed on the 2nd to 3rd day of the menstrual cycle. At this time, the thickness of the endometrium was not greater than 6 mm. Seven days later, after ruling out abnormalities such as functional ovarian cysts and determining the endometrial thickness during the previous ovulation period, 46 mg/d estradiol valerate (Busjale, Bayer Medical & Health Co., Ltd.) was administered. For vaginal ultrasound, if the endometrium was well developed, the initial dose was administered for another 3 to 5 days. If the endometrial thickness was less than 7 mm after reexamination, the maximum oral supplement dosage (8 mg/d) was administered for 3-5 days. Estrogen was administered for at least 10 days. For patients with an endometrial thickness >7 mm and an E_2_ level ≥100 ng/L, the duration of P-induced endometrial transformation was set according to each patient’s schedule. Progesterone was used to transform the endometrium via the same procedure as that used previously described.

#### Downregulation + artificial cycle

This cycle was applied in patients with endometriosis. After vaginal ultrasonography was performed on the 2nd to 3rd days of the menstrual cycle, 3.75 mg of triptorelin acetate was given by injection if there were no obvious contraindications; vaginal ultrasonography was repeated 28 days later, and the same artificial cycle for endometrial preparation and transformation regimens were used if there were no abnormalities.

### Transvaginal ultrasonography for detecting the uterine artery PI

Vaginal ultrasound was performed on all participants at a fixed time (10-12 AM each day) on the day of inner membrane transformation for frozen–thawed embryos. A GE VOLUSONE6 color Doppler ultrasonic diagnostic instrument was used, and the probe frequency in the cavity was set to 5~9 MHz. After each patient emptied her bladder, she was placed in the bladder lithotomy position, and a vaginal probe was placed inside the her vagina using a disposable condom. The uterus and the inner cervix were displayed in the median sagittal view, or the inner cervix was displayed in the transverse view. The uterine artery was displayed by moving the probe (the inner cervix was horizontal and opened to the lateral side by 1-2 cm). During the observation of uterine artery blood flow, the color Doppler gain was adjusted to be moderate, the scanning speed was adjusted, the sampling volume was 2 mm, and the sampling line was as consistent as possible with the direction of blood flow (angle < 50°). More than three continuous stable and consistent spectral images of uterine artery blood flow were obtained, and the blood flow parameter values of both uterine arteries were automatically recorded. The average value of the bilateral uterine artery PI was calculated.

### Observation indicators and definitions

Clinical pregnancy was defined as the presence of one or more pregnancy sacs on ultrasound and diagnosed as a clinical pregnancy, including normal intrauterine pregnancy, ectopic pregnancy, or extrauterine pregnancy. Pregnancy loss was defined as the presence of a pregnancy sac without fetal heart motion. The clinical pregnancy rate was calculated as the number of clinical pregnancies/the number of total transplant cycles×100%.

### Statistical analysis

All the statistical analyses were performed using SPSS software, version 22.0. Continuous variables are expressed as the mean ± standard deviation (SD), and the rank-sum test was used to compare quantitative data among groups. The adoption rate of qualitative data (%) indicated that the qualitative data among multiple groups were tested. Binary logistic regression analysis was used to identify factors influencing clinical outcomes. The inclusion factors included maternal age (continuous variable), duration of infertility (continuous variable), type of infertility (categorical variable), infertility diagnosis (categorical variable), endometrial preparation protocol (categorical variable), AMH (continuous variable), endometrial thickness (continuous variable), number of transferred embryos (continuous variable), embryo stage at transfer (categorical variable) and the mean PI (categorical variable). Propensity score matching (PSM) was used to correct for bias caused by the uneven distribution of the baseline data of the study subjects; PSM was performed at a ratio of 1:1. The matching data included age, infertility duration, body mass index, infertility type, infertility cause, baseline follicle-stimulating hormone (FSH) level, antral follicle count (AFC), anti-Müllerian hormone (AMH) level, number of transferred embryos, morphology of the transferred embryos, endometrial thickness, medication regimen and clinical pregnancy rate. The caliper value was 0.02.

All patient data and follow-up information were obtained from the electronic medical records system of the reproductive center of the Third Affiliated Hospital of Zhengzhou University. A two-sided P value <0.05 was considered to indicate statistical significance.

## Results

### Study population

**Figure 1:**
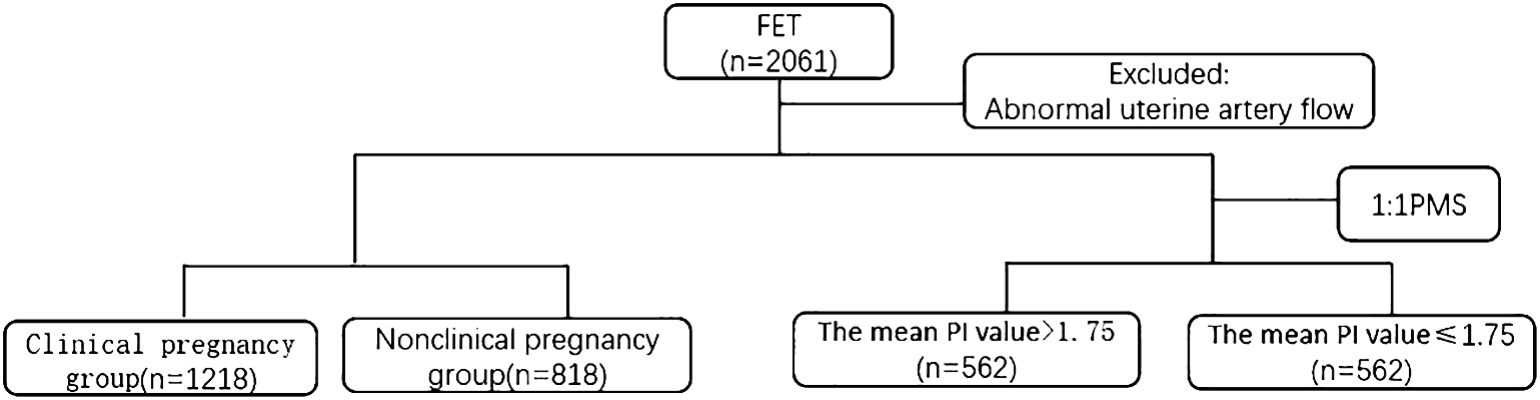
Flow chart.

In total, 2,061 patients who underwent only one FET were included, and 25 of them had abnormal uterine artery blood flow (blood signal loss in the early diastolic period, reverse diastolic blood flow in the early diastolic period, blood flow loss in the late diastolic period or blood flow loss in the diastolic period); moreover, the PI value was infinite. Among the patients, 12 had clinical pregnancies, and the clinical pregnancy rate was 48%. After exclusions, 2,036 patients were included; 1,218 had clinical pregnancies, and the clinical pregnancy rate was 59.8%. The subjects were divided into a clinical pregnancy group and a nonclinical pregnancy group according to pregnancy outcome. A multivariate logistic regression model was used to analyze the factors affecting the clinical pregnancy rate. The receiver operating characteristic (ROC) curve was used to determine the optimal mean PI cutoff value of 1.75. After 1:1 PSM, 562 patients were included. For statistical description and analysis, the patients were divided into two groups: a group with a mean PI > 1.75 and a group with a mean PI ≤ 1.75. The study flow chart is shown in **Figure 1**.

### Baseline characteristics

There were significant differences in age, infertility type, infertility factors, FSH and AMH levels, AFC, number of transferred embryos and stage of transferred embryos. There were no significant differences in the mean PI value or other baseline characteristics (**Table 1**).

**Table 1:** Comparison of patient basic information.

	Clinical pregnancy group(n =1218)	Nonclinical pregnancy group(n =818)	P value
Maternal age (years)	30.6 ± 3.9	31.7 ± 4.1	<0.01
Duration of infertility (years)	3.3 ± 2.6	3.4 ± 2.8	0.25
Body mass index (kg/m^2^)	24.1 ± 3.3	24.1 ± 3.2	0.68
Type of infertility			0.04
Primary infertility	46.6% (568/1218)	42.1% (344/818)	
Secondary infertility	53.4% (650/1218)	57.9% (474/818)	
Infertility diagnosis			0.47
Tubal factor	35.6% (434/1218)	38.0% (311/818)	
Ovulation disorder	12.7% (155/1218)	13.4% (110/818)	
Male factor	21.8% (265/1218)	18.6% (152/818)	
Male+female factors	20.8% (253/1218)	21.3% (174/818)	
Unknown cause	9.1% (111/1218)	8.7% (71/818)	
Basal FSH (U/L)	5.9 ± 2.5	6.2 ± 3.0	0.04
AFC	19.7 ± 8.0	17.7 ± 8.1	<0.01
AMH (pmol/L)	29.0 ± 25.0	24.6 ± 22.2	<0.01
Number of transferred embryos	1.4 ± 0.5	1.5 ± 0.5	0.01
Embryo stage at transfer			<0.01
Blastocysts Cleavage-stage embryos	66.0% (804/1218) 34.0% (414/1218)	49.3% (403/818) 50.7% (415/818)	
Endometrial Thickness (mm)	9.3 ± 1.5	9.2 ± 1.5	0.16
Endometrial preparation protocol			0.66
Downregulation +artificial cycle	5.8% (71/1218)	6.7% (55/818)	
Artificial cycle	45.5% (554/1218)	43.9% (359/818)	
Natural cycle	37.5% (457/1218)	39.1% (320/818)	
Induced ovulation cycle The mean PI value	11.2% (136/1218) 2.0 ± 0.3	10.3% (84/818) 2.0 ± 0.4	0.10

The data are presented as the % (n/N) for categorical variables.

### Binary logistic regression analysis was used to correct for the factors affecting the clinical pregnancy rate

To reduce the interference from the confounding factors, binary logistic regression analysis was performed. The included variables were age, infertility type, infertility cause, infertility duration, endometrial preparation protocol, AMH level, endometrial thickness, number of transferred embryos, stage of transferred embryos, and mean PI. The results of the multivariate analysis showed that maternal age (AOR=0.95, 95% CI=0.93-0.98, P<0.01), AMH level (AOR=1.00, 95% CI=1.00-1.01, P=0.045), number of transferred embryos (AOR=1.98, 95% CI=1.47-2.70, P<0.01), and type of transferred embryo (AOR=3.10, 95% CI=2.27-4.23, P<0.01) were independent factors influencing the clinical pregnancy rate. The mean PI (AOR=0.85, 95% CI=0.70-1.05; P=0.13) was not an independent factor influencing the clinical pregnancy rate (**Table 2**).

**Table 2:** Binary logistic regression model analysis of the effect of the clinical pregnancy rate on frozen-thawed embryo transfer.

	AOR	95% CI	P Value
Maternal age (years)	0.95	0.93-0.98	<0.01
Type of infertility (primary/secondary)	0.99	0.80-1.23	0.93
Infertility diagnosis(tubal/ovulation disorders/male/others)	1.03	0.96-1.10	0.46
Duration of infertility (years)	1.00	0.96-1.03	0.80
Endometrial preparation protocol Induced ovulation cycle Artificial cycle Natural cycle Downregulation+artificial cycle	1.00(Ref) 1.12 1.06 1.06	0.70 0.77 0.77	0.97 1.79 1.47 1.46
AMH (pmol/L)	1.00	1.00-1.01	0.045
Endometrial Thickness	1.05	0.98-1.11	0.16
Number of transferred embryos	1.98	1.46-2.70	<0.01
Embryo stage at transfer(blastocysts/cleavage-stage embryos)	3.10	2.27-4.23	<0.01
The mean PI	0.85	0.70-1.05	0.13

AOR, adjusted odds ratio; CI, confidence interval.

The mean bilateral uterine artery PI cutoff value was 1.75, which was used to divide the patients into two groups: the mean PI value > 1.75 group and the mean PI value ≤1.75 group. After 1:1 PSM, 562 patients were included for statistical description and analysis. There were no significant differences in age, infertility duration, body mass index, infertility type, infertility factors, baseline FSH level, AFC, AMH level, number of transferred embryos, embryo stage, endometrial thickness, endometrial preparation regimen or clinical pregnancy rate between the two groups (P>0.05) (**Table 3**).

**Table 3:** Comparison of basic data between the group with a mean PI > 1.75 and the group with a mean PI ≤ 1.75.

	Mean PI >1.75 (n =562)	Mean PI ≤1.75 (n =562)	P Value
Maternal age (years)	31.6 ± 4.0	31.56 ± 4.0	0.99
Duration of infertility (years)	3.2 ± 2.8	3.3 ± 2.8	0.73
Body mass index (kg/m2)	23.8 ± 3.3	23.9 ± 3.2	0.46
Type of infertility			0.54
Primary infertility	40.2 (226/562)	38.4% (216/562)	
Secondary infertility	59.8 (336/562)	61.6% (346/562)	
Infertility diagnosis			0.47
Tubal factor	37.7 (212/562)	38.1% (214/562)	
Ovulation disorder	11.7 (66/562)	15.1% (85/562)	
Male factor	21.0 (118/562)	18.5% (104/562)	
Male+female factors	20.6 (116/562)	9.1% (51/562)	
Unknown cause	8.9 (50/562)	8.7% (71/818)	
Basal FSH (U/L)	6.0 ± 2.8	6.0 ± 2.8	0.90
AFC	18.4 ± 7.9	18.7 ± 8.4	0.51
AMH (pmol/L)	27.2 ± 23.9	27.2 ± 24.8	0.99
Number of transferred embryos	1.4 ± 0.5	1.5 ± 0.5	0.40
Embryo stage at transfer			0.72
Blastocysts Cleavage-stage embryos	57.1 (321/562) 42.9 (241/562)	56.0% (403/562) 44.0% (415/562)	
Endometrial Thickness (mm)	9.3 ± 1.5	9.3 ± 1.6	0.81
Endometrial preparation protocol			0.14
Downregulation+artificial cycle	7.3 (41/562)	8.5% (48/562)	
Artificial cycle	39.1 (220/562)	44.8% (252/562)	
Natural cycle	41.5(233/562)	35.9% (202/562)	
Induced ovulation cycle Clinical pregnancy rate	12.1 (68/562) 56.2 (316/562)	10.7% (60/562) 56.0% (315/562)	0.10

The data are presented as the % (n/N) for categorical variables.

## Discussion

The uterine artery PI is currently the most commonly used indicator that can best reflect the blood flow parameters of the uterine artery (6–9). In this study, when the mean PI values of the clinical pregnancy group and the nonclinical pregnancy group were compared, no statistical significance was observed. Binary logistic regression analysis revealed that age, AMH level, number of transferred embryos and stage of transferred embryos were independent factors influencing the clinical pregnancy rate, which is consistent with the findings of many other studies (10). However, the mean PI in this study was not an independent influencing factor of the clinical pregnancy rate.

There are many studies on uterine artery blood flow parameters during fresh embryo transfer cycles but few on this topic during FET cycles (7, 11, 12). Numerous studies have presented different results. Moreover, uterine artery blood flow parameters cannot predict pregnancy outcomes well (12). A study showed that the mean PI of the uterine artery in the nonpregnant group was 2.31 ± 0.48, and the mean PI in the pregnant group was 2.22 ± 0.36, with a P value of 0.35, indicating no significant difference (13). Other studies with the opposite view (14–18) have shown that an increase in uterine artery blood flow resistance leads to a decrease in endometrial vascularization, thus affecting blood circulation between the mother and fetus and uterine blood flow, thus affecting pregnancy outcome. The uterine artery resistance index (RI) and PI in the fresh cycle group were significantly lower than those in the nonimplantation group (19). Endometrial thickness and the blood flow indices of the endometrium and uterus measured by transvaginal sonography are not effective predictors of pregnancy outcomes in FET cycles (20).

Previous studies have shown that the cutoff PI for implantation failure ranges from 2.5 to 3.3 (16, 17). In this study, the truncation value of the mean bilateral uterine artery PI was 1.75, and PSM at a ratio of 1:1 was used to stratify patients into a mean PI > 1.75 group and a mean PI ≤ 1.75 group. There were no significant differences in the various parameters between the two groups, indicating, once again, that the mean PI cannot predict clinical pregnancy outcomes well.

This study has certain limitations. First, this was a retrospective cohort study, which may be influenced by confounding factors. However, binary logistic regression was adopted in this study to correct for the interference of confounding factors as much as possible. Second, in the process of measuring the uterine artery PI, relatively large fluctuations were found in some patients, but no relevant literature was found to explain the underlying reasons; this may also explain why the parameters of uterine artery flow could not predict pregnancy outcome well. Third, the limitation of this study is that only patients aged <40 years were recruited; for the above reasons, the results may not be generalizable to older patients. Finally, the previous literature has shown that the loss of blood flow in the early diastolic period, that is, the PI, is infinite, and the pregnancy success rate is extremely low. However, the data in this study showed that although the clinical pregnancy rate of 48% of patients with blood flow loss in the early diastolic period was slightly lower than that of 59.8% of patients without blood flow loss, the error was relatively large due to the small sample size. Thus, further increasing the sample size is necessary for additional research and analysis.

## Conclusions

On the basis of our study, although transvaginal Doppler ultrasonography is an economical, convenient and practical method for monitoring uterine artery blood flow, the mean PI on the day of endometrial transformation in patients undergoing FET cycles does not predict pregnancy outcomes well.

## Data availability statement

The raw data supporting the conclusions of this article will be made available by the authors, without undue reservation.

## Ethics statement

The studies involving humans were approved by Third Affiliated Hospital of Zhengzhou University (2022-204-01). The studies were conducted in accordance with the local legislation and institutional requirements. Written informed consent for participation was not required from the participants or the participants’ legal guardians/next of kin because This was a retrospective cohort study.

## Author contributions

LL: Writing – original draft, Conceptualization, Data curation, Formal analysis. MD: Data curation, Writing – review & editing, Formal analysis. SW: Methodology, Writing – review & editing. CW: Resources, Writing – review & editing. PK: Supervision, Writing – review & editing. JZ: Supervision, Writing – review & editing. YG: Funding acquisition, Supervision, Writing – review & editing.
